# Isolation and molecular identification of wild Newcastle disease virus isolated from broiler farms of Diyala Province, Iraq

**DOI:** 10.14202/vetworld.2020.33-39

**Published:** 2020-01-04

**Authors:** Amer Khazaal Alazawy, Karim Sadun Al Ajeeli

**Affiliations:** Department of Microbiology, College of Veterinary Medicine, University of Diyala, Diyala, Iraq

**Keywords:** Diyala Province, Newcastle disease virus, pathogenicity, real-time polymerase chain reaction

## Abstract

**Background and Aim:**

Newcastle disease virus (NDV) remains a major viral disease of poultry. The morbidity and mortality rates of chickens vaccinated with NDV in broiler farms in Diyala Province were 100% and 80%, respectively, rates due to suspected infection with the highly virulent NDV. The present study aimed to isolate and identify the NDV virus and evaluate its pathogenicity in infected broiler chickens at poultry farms.

**Materials and Methods:**

Broiler chickens at two commercial poultry farms were suspected of being infected with virulent NDV due to high mortality rates. Virus isolated from samples of intestinal tissues, lungs, trachea, spleen, kidneys, and air sacs was adapted in the allantoic cavity of embryonated specific-pathogen-free (SPF) chicken eggs. The NDV pathotype was determined based on the mean death time (MDT) in eggs as well as the intracerebral pathogenicity index (ICPI) and intravenous pathogenicity index pathogenicity indexes of the isolated samples. Broilers were experimentally infected by inoculation with fluids collected from the allantoic cavities of 60 broilers aged 35 days. Serological and molecular tests were followed by enzyme-linked immunosorbent assay to determine levels of anti-NDV immunoglobulin G, and isolates were identified using a hyperimmune (HI) test and real-time polymerase chain reaction (RT-PCR).

**Results:**

Suspected and isolated NDV field samples propagated in the allantoic cavity of 10-day-old fertile SPF chickens were NDV positive in the rapid hemagglutination test within a few seconds. Pathogenicity indices and MDT showed that the isolated NDV was viscerotropic and velogenic. The virus was identified as NDV by the HI test using specific anti-LaSota HI serum and RT-PCR with specific primers and probes. Propagation of the virus in the allantoic cavity of embryonated hen eggs produced a viral titer of 10^9.5^ EID_50_/0.1 mL.

**Conclusion:**

The virus isolated from broiler chicken farms in Diyala Province, Iraq, was viscerotropic and velogenic according to the pathogenicity indices and RT-PCR. The isolated NDV caused 100% morbidity and 90% mortality in NDV-vaccinated and experimentally infected broiler chickens.

## Introduction

Newcastle disease (ND) is regarded as one of the most highly contagious and fatal viral diseases of poultry and is a major worldwide concern [1-3]. About 250 avian species in 27 of 50 orders of birds are reportedly susceptible to natural or experimental infection with ND virus (NDV), and many more species might be susceptible but remain identified [4-6]. The World Organization for Animal Health (OIE) has classified NDV infection as a notifiable disease. The first reports of the disease in poultry originated from Java, Indonesia, and Newcastle upon Tyne in England during 1926, but the disease is now distributed worldwide [7]. The virus has been formally recognized as avian paramyxovirus 1 (APMV-1) and classified within the genus *Avulavirus* of the family *Paramyxoviridae* and the order *Mononegavirales* [8-10]. It is an enveloped, linear, non-segmented, and negative-sense single-stranded RNA virus [11] with a genome containing six genes with 15,186 nucleotides. These genes include nucleoprotein (NP), phosphoprotein (P), matrix protein (M), fusion protein (F), hemagglutinin-neuraminidase (HN) protein, and large protein (L) 3′-NP-P-M-F-HN-L-5′ [12]. The virulence of NDV strains varies greatly according to the host. Among poultry, chickens and turkeys are the most susceptible; ducks and geese are the least susceptible and are generally considered as NDV carriers that are resistant to NDV strains, even strains that are the most virulent for chickens [13].

The occurrence of NDV outbreaks in flocks vaccinated against NDV suggests the emergence of new virulent strains that lead to high morbidity and mortality rates among affected birds and considerable associated economic loss. Such evidence indicates that circulating NDV strains should be continuously evaluated to determine management strategies to reduce loss due to such infections. The pathotypes of NDV strains are viscerotropic velogenic, neurotropic velogenic, mesogenic, lentogenic, and asymptomatic [14]. The pathotypes of NDV can be determined using the intracerebral pathogenicity index (ICPI) in 1-day-old chicks, the intravenous pathogenicity index (IVPI) in 6-week-old chickens, and the mean death time (MDT) of embryonated chicken eggs (ECE) [15-16]

The study aimed to isolate virulent NDV from specimens obtained from chickens with suspected virulent NDV infection during a local outbreak in Diyala Province, Iraq, identify the isolated virus using serological hyperimmune (HI) tests, enzyme-linked immunosorbent assay (ELISA), and real-time polymerase chain reaction (RT-PCR), and determine its pathogenicity.

## Materials and Methods

### Ethical approval

The Scientific Ethical Committee of College of Veterinary Medicine, Uinversity of Diyala, Iraq approved this study (Approval no: Vet Medicine (20); January 2019, A and K).

### Background

Two commercial poultry farms in different areas of Diyala Province were randomly selected during the late summer of 2018. Each farm contained about 8000 4-week-old broilers with suspected NDV infection based on the characteristic clinical findings of severe greenish diarrhea, depression, and eye swelling, and postmortem lesions associated with NDV infection. The mortality rate started at 15-25% and reached 80% within a few days when 600 birds/day died daily at each farm. The clinical diagnostic postmortem findings of ND revealed severe hemorrhagic lesions in the intestinal tract, petechial hemorrhages in the mucosa of the proventriculus, severe inflammation of the trachea, thickening of the air sacs, and hemorrhage in the spleen. The broiler chickens in these farms were vaccinated twice (at 2 and 3 weeks of age) with LaSota NDV vaccine Nobilis ND Clone 30 (Intervet Inc., Hillsboro, DE, USA) with at least 10^6^ EID_50_ of the NDV LaSota strain. The two broiler farms were well managed and ventilated, equipped with all necessary, feeders, thermometers, and water and light sources. All birds were given food and water *ad libitum*. Both farms were separated from other broiler farms by a considerable distance.

### Sample collection and processing

Samples were collected under aseptic conditions from the intestinal tissues, lungs, trachea, spleen, kidneys, and air sacs of 10-15 diseased and/or freshly-dead chickens from each farm for viral isolation. The samples were immediately transferred to the virology laboratory at the College of Veterinary Medicine, University of Diyala. Under a laminar flow cabinet (Labnet International Inc., Edison, NJ, USA) small pieces of tissues (2 g each) were cut using sterile forceps and scissors, ground with sterile sand in a sterile mortar and pestle, then transferred to 1500-mL sterile test tubes containing phosphate-buffered saline (PBS; 9 mL), pH 7.4, containing penicillin (1,000 IU/mL), streptomycin (10 mg/mL), gentamycin (250 mg), and anti-PPLO (6 mg/mL). The suspension was placed at 4°C for two h and clarified by centrifugation at 2000 rpm for 10 min at 4°C. Suspensions of homogenized tissue samples were clarified by centrifugation at 3000 rpm at 4°C for 10 min, and then the supernatants were transferred to new sterile test tubes, labeled, and either used immediately or stored at −80°C.

### Virus isolation

The virus was isolated as described in previous studies [17-19]. In brief, six hen eggs were inoculated with the virus and four served as controls. The allantoic cavities of 10-day-old specific-pathogen-free (SPF) ECE were inoculated with sample supernatants (200 µL). Control embryonated eggs were inoculated in the same method with sterile normal saline.

All opened shells were closed with wax and the eggs were incubated at 37°C for 5-7 days and checked daily for embryo viability. Embryos that died within 24 h post-infection (PI) were excluded and regarded as death due to non-specific causes. All embryos that died after 24 h or survived until the end of the incubation period were chilled at 4°C overnight, and then allantoic fluids were assessed slide hemagglutination (HA) tests against one drop of 4% avian red blood cell (RBC) in normal saline and one drop of inoculated allantoic fluid on glass slides. Positive HA allantoic fluid was collected, pooled, divided into portions and stored in sterile screw-capped vials at −80°C.

### Preparation of HI serum

HI serum against LaSota NDV vaccine strain was prepared in rabbits as described [20]. After one week post–inoculation (PI), the inoculated rabbits were bled and serum was separated by centrifugation at 4000 rpm for 5 min at 4°C. Serum supernatant was distributed in 2-mL vials and stored at −30°C.

### Serological identification of NDV

Plate HA inhibition (HI) tests proceeded as follows. Four HA units/0.1 mL of test allantoic fluids were reacted against HI serum prepared in rabbits and serially diluted two-fold in PBS. Thereafter, an equal volume of 4% chicken RBC in PBS was added. The HI endpoint was determined as the reciprocal of the highest dilution of serum that caused 100% HA inhibition.

### Pathotyping of NDV

The NDV pathotype was determined by MDT and pathogenicity indices as follows.

#### MDT in eggs

The MDT was determined by inoculating 9-11-day-old embryonated SPF hen eggs as described [21] with ten-fold serial dilutions of NDV field isolates, then incubating them at 37°C until the embryos died. Velogenic and lentogenic strains of NDV caused embryo death within 60 and >90 h, respectively. We considered that any NDV strain that caused embryo death of >60 but <90 h was mesogenic.

#### ICPI

The ICPI was determined as described [16]. Briefly, fresh infective allantoic fluid was diluted 1:10 in sterile isotonic saline without additives or antibiotics. The caudal areas of the brains of 10 chicks hatched from the eggs of an SPF flock were intracerebrally inoculated with 0.05 mL of 1:10 diluted fresh allantoic fluid using a sterile tuberculin syringe and examined every 24 h for 8 days.

#### IVPI

Fresh infective allantoic fluid containing virus (0.1 mL) diluted 1:10 in sterile isotonic saline was intravenously injected into 10 6-week-old SPF chickens, then the birds were assessed at 24-h interval for 10 days [16].

### Experimental infection

The virulence and the infectivity of stock virus NDV were determined in susceptible broiler chicks as follows. Ross 308 broiler chicks aged 81 days obtained from a commercial hatchery were provided with commercial pelleted feed and water *ad libitum*. The chicks were not given any antibiotics and were vaccinated only against NDV (Clone30/Holland) suspended in drinking water at intervals of 7 days until they reached 21 days of age. We inoculated 60 6-week-old chickens with local NDV isolate and placed 20 control birds as far from the infected chickens as possible to avoid transmission of the isolate. The anti-NDV immunoglobulin G (IgG) titer in the blood of these birds was determined at the ages of 3, 18, and 25 days using ProFLOK^®^ PLUS NDV ELISA kits (Synbiotics Corporation, Platte City, MO, USA).

The experimental chickens were nasally and orally inoculated with 0.1 mL of 1:10-diluted stock virus. The control chicks were similarly inoculated with allantoic fluid from non-infected embryonated control eggs diluted 1:10 in sterile normal saline. All chickens were assessed daily for clinical signs and mortality.

Freshly dead and diseased chickens were evaluated postmortem, and tissue samples were collected from the lungs, trachea, spleen, liver, kidneys, intestine, and brain for virus isolation and molecular identification using RT-PCR.

### RNA extraction

Viral RNA was extracted from tissue samples using Patho Gene-Spin DNA/RNA Extraction Kits (iNtRON Biotechnology Inc., Seongnam-Si, South Korea) as described by the manufacturer. We used one-step RT-PCR kits (Qiagen, Hilden, Germany) for real-time reverse-transcription PCR (RRT-PCR) as described [22] using 25 µL reaction volumes.

### M gene primers and probe

The highly conserved region of the M genome was amplified as described [23] using the forward APMV 1F and reverse APMV1R primers and probe (Alpha DNA Laboratories, Montreal, QC, Canada), as shown in Table-1.

**Table-1:** Primers used in real-time polymerase chain reaction.

Probe and primers	Sequence
Probe:HEX APMV1-LNA	(5′ HEX-gggaCrGChTgCtatCc-BHQ3′)
Primer forward:APMV1F	(5′AGTGATGTGCTCGGACCTTC3′)
Primer reverse:APMV1R	(5′ CCTGAGGAGAGGCATTTGCTA 3′)

APMV1=Avian paramyxovirus 1

The M gene was amplified in a reaction mix containing 1 µL of enzyme mix comprising Hot Start Taq polymerase, reverse transcriptase and 5 µL of buffer supplied with the RT-PCR kits, 10 pmol each of reverse and forward primers, 6 pmol of probe and 2× RT-PCR master mix. Table-2 shows the RT-PCR amplification conditions that were identical to those provided by the manufacturer.

**Table-2:** Amplification program for real-time polymerase chain reaction assay.

No.	Step	Temperature	Time
1.	Reverse transcriptase	50°C	20 min
2.	Initial denaturation	95°C	15 min
3.	Denaturation	94°C	45 s
4.	Annealing	60°C	45 s

The cycling conditions for the APMV-1 matrix primers consisted of 40 cycles

## Results

### Isolation and identification of NDV

Field samples with suspected and isolated NDV propagated in the allantoic cavities of 10-day-old fertile SPF chicks through were positive in the rapid HA tests within a few seconds. The microplate HA test to titrate the virus showed a titer of 1024 HAU/0.1 mL. The titer of the field isolate identified by HI using LaSota HI serum was 2^16^ HIU/0.1 mL of stock serum.

### Viral pathogenicity

The pathogenicity of virus isolates was determined from MDT findings and pathogenicity indices. The MDT test showed that the 10^−9^ diluted virus killed all inoculated embryonated eggs within 60 h. The EID_50_ of the isolated virus was 10^−9.5^ EID_50_/0.1 mL of stock virus. The ICPI and IVPI showed that all embryonated eggs were killed within 60 h PI. These indices showed that the NDV isolate was velogenic.

### Detection of the virus in samples from experimentally infected chickens

**Figure-1:**
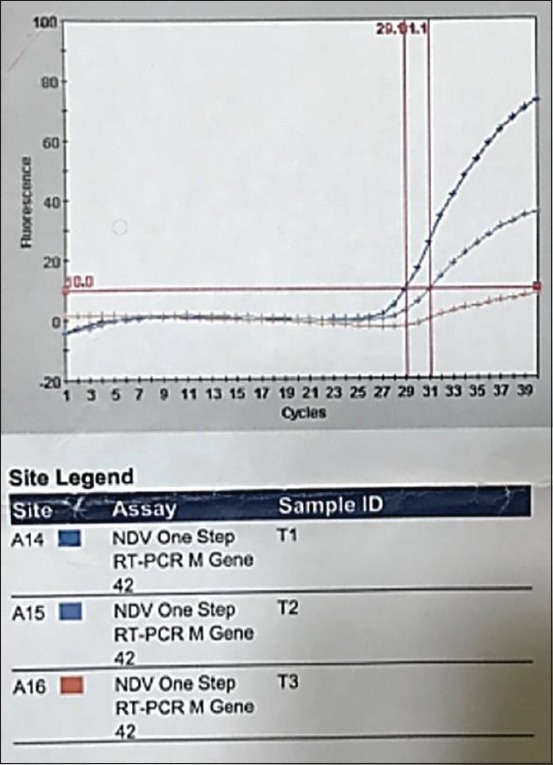
Smart cycle fluorography is illustrating the detection real-time reverse-transcription polymerase chain reaction for viral nucleic acid for the avian paramyxovirus 1 matrix gene of the sample from experimentally infected chickens.

Clinical signs in challenged broilers started to appear from 2 days post-infection as mild nasal discharge, depression, and tachypnea. The conjunctivae were also swollen with red spots on lymphoid patches of the lower eyelids, and the birds also appeared inappetent. Clinical signs rapidly developed thereafter, and the infected birds had greenish diarrhea, severe nasal discharge, and depression. Nerve signs in most infected birds were characterized by leg paralysis and head torticollis. The mortality rate reached 90% when 54 of 60 birds died. None of these clinical symptoms were evident among the control birds, and all of them survived until the end of the experiment. Three birds were randomly selected from the NDV-infected group for molecular detection using RT-PCR. Two samples were positive in the APMV-1 M gene procedure. The mean number of cycle thresholds (Ct) for Sample A 14 that was positive by RT-PCR was 29.1 compared with the second Sample A 15, which was 31.1 (Figure-1) and the negative sample, a 16, which was 0 (Table-3). The Ct value and the endpoint (Endpt) inversely correlated in the two positive samples. The efficiency of the RRT-PCR reaction for doubling the M gene at the exponential phase was 100%, which indicated that the amplification was stable.

**Table-3:** The results of real-time polymerase chain reaction performed on three randomly selected samples of Newcastle disease virus experimentally infected birds.

Site ID	Sample ID	Assay result	Cycle thresholds	Endpt
A 14	T1	Positive	29.1	72
A 15	T2	Positive	31.1	35
A 16	T3	Negative	0	8

### Maternal and post-vaccination NDV antibodies

The mean IgG antibody level at the age 3 of days (maternal antibodies) was 7815.44 (Table-4). This value was obviously reduced at the age 18 days and after a second vaccination to 2080.55, as well as at the age of 25 days and after the third vaccination to 504.33 (Table-4).

**Table-4:** Antibody immunoglobulin G levels in broilers before Newcastle disease virus experimental infection.

Age of broilers	No. of samples	Mean of Ab	GMT	Standard deviation	Standard error of mean	CV%
Maternal Ab at 3 days	18	7815.44^a^	7731	1179.75	278.07	10.35
At 18 days	18	2080.55^b^	1944	721.02	169.94	24.55
At 25 days	18	504.33^c^	146	392.75	92.57	25.85
Sig.		**				
p-value		1E-9				

Ab=Antibodies, GMT=Geometric mean, %CV=Coefficient of variation,

** refer to highly significant effects in analysis of variance. Means have different letters refer to significant differences at p≤0.05

### Postmortem findings in experimentally infected birds

**Figure-2:**
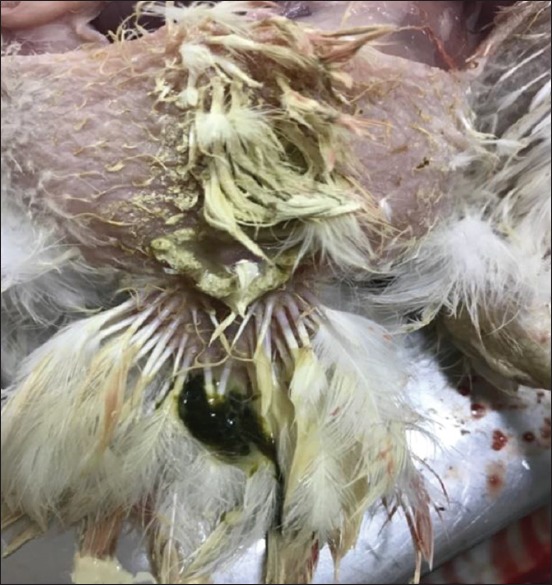
The inoculated chickens showed greenish diarrhea surrounding the cloaca (arrow).

**Figure-3:**
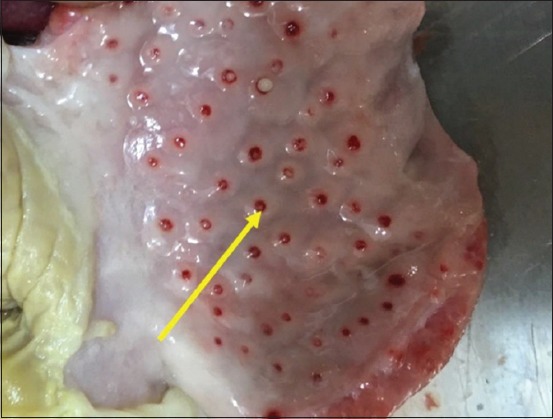
Experimental infection of chickens showed petechiae and small ecchymoses on the tips of proventricular glands (arrow).

**Figure-4:**
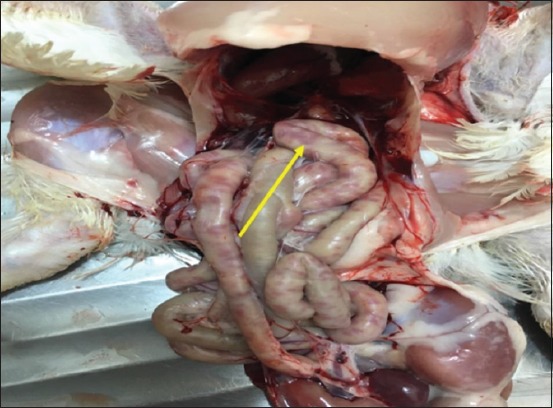
The intestine of infected chicken showed clear hemorrhagic foci that appeared dark red from outside vision (arrow).

**Figure-5:**
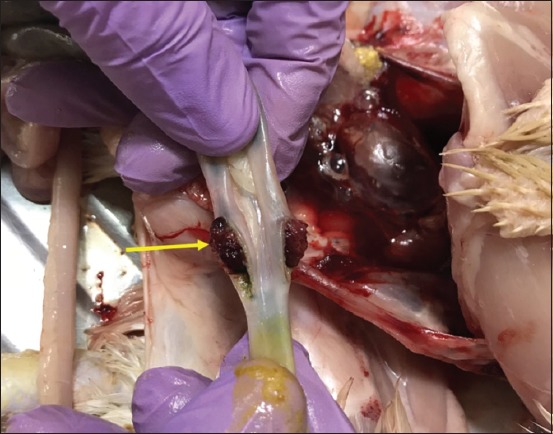
Enlargement and hemorrhages of cecal tonsils (arrow).

Most infected and dead birds had clear greenish diarrhea on their cloacal area that also covered feathers in this region (Figure-2). Opisthotonus, congestion in the lungs, trachea, liver and spleen, air sacculitis, head swelling, enteritis, and conjunctival swelling with red spots on the lymphoid patches of the lower eyelids were also evident. Postmortem examinations of infected birds showed pinpoint hemorrhages at the tips of the proventricular glands (Figure-3), hemorrhagic ulcers in the intestinal wall (Figure-4), and enlarged cecal tonsils that appeared edematous, bloody, and necrotized (Figure-5). The lungs with air sacculitis and the trachea, liver, and spleen were all congested. All samples collected from such birds were positive for virus in embryonated eggs and were identified in HI tests using anti-LaSota HI serum. The morbidity rate reached 90% in the experimental birds and mortality rates ranged from 10% to 90%, with death occurring within 24-72 h after the onset of clinical signs.

## Discussion

ND one of the leading threats to the health and welfare of poultry globally. The OIE has classified it as a listed disease, which requires that outbreaks of mesogenic or velogenic ND must be reported to the OIE [17].

Despite widely implemented vaccination, ND has remained endemic in many parts of the world, including developed countries. Infection with NDV occasionally emerges even in countries that are considered free of this disease [24].

The first finding of NDV in Iraq was during 1968 when it was isolated from infected chickens at Abu Ghraib, and designated as AG68 [25]. ND in Iraq has since been regarded as endemic. The present study isolated and identified NDV from samples collected from naturally infected broilers. The findings of NDV isolation in the present study support the findings of others [3,26] who reported that NDV isolated and identified from samples of chickens with suspected infection starts to cause embryonic mortality from 48 h PI and the death of all embryos within 120 h PI.

The present pathogenicity indices indicated that the isolated virus was velogenic. This finding was contrary to those of the previous study [3], in which NDV strains with ICPI values of 1.5-2.0, 1.0-1.5, and 0.2-0.5 were classified as velogenic, mesogenic, and lentogenic, respectively. Another study found that selected NDV field isolates with ICPI values of 1.55-1.79 were consistent with velogenic NDV isolates [27].

The one-step RRT-PCR in the present study was highly sensitive and reliably detected the M gene of RNA APMI, which is in agreement with the findings of the previous studies [23] in which the sensitivity of the RRT-PCR to detect viral genome consistently varied, although this provided ease of use and reduced opportunities for cross-contamination. In contrast, one study found that values were 100-fold higher using a highly sensitive RT-PCR assay for NDV involving two-step, compared with one-step viral RNA amplification [28]. The NDV specific primers used in direct RT-PCR for the genomic detection of NDV were equally sensitive and specific for the RNA extracted from clinical and postmortem samples. The present findings were in line with a study [23] showing that the one-step technique was more sensitive because the APMV-1 (M gene) could be consistently detected earlier during infection using a specific primer-probe set than another NDV gene.

A previous study [29] found that a high Ct value (above 38 cycles) indicated cross-contamination of amplified nucleic acid or an insufficient amount of nucleic acid for amplification. Similarly, one study negatively associated the quantitative Ct value with the (log) concentration of detected nucleic acids; for example, a high Ct value reflects a low target concentration and vice versa [30]. The RRT-PCR in the present study generated Ct values ranging from 0 to 31.1.

High serum IgG values in 3-day-old birds might be attributable to the vertical transmission of maternal antibodies to progeny against certain pathogens, as this is a crucial means of protection up to a certain age [31]. The reduction in IgG antibody levels at 2 weeks post-vaccination was significant (p≤0.05) compared with that at the age of 3 days (maternal antibody titer). This was in agreement with the finding that ~ 30% of the IgG and 1% of the immunoglobulin M and immunoglobulin A antibodies present in hen plasma as maternal antibodies that passively transfer across egg yolks from mother to offspring play a key role in protecting hatching chicks against pathogenic attacks and then they decline [31-33]. Antibody titers gradually fall from peak to low levels at post-vaccination weeks 3 and 4 [32]. In contrast, protection after vaccination can be reflected by low or undetectable levels of antibodies [34]. Our findings also agreed with the fact that vaccinating chicks while maternal antibody levels remain high results in vaccine failure, through neutralization of the live vaccine and maternal antibodies play roles in determining the responses of chicks to early vaccination [35]. Clinical signs characterized by respiratory and nervous system signs, severe greenish diarrhea, and severe nasal discharge and depression in the present study appeared 2-3 days post-inoculation in birds that were experimentally infected at 42 days of age. Compared with the previous studies, isolated velogenic NDV strains are highly pathogenic to all susceptible species and cause high mortality with typical clinical signs [36].

The findings of high morbidity and mortality rates from our field experience and the present study indicate that vaccination does not always increase protective immunity against NDV.

Although vaccination provides good protection against clinical disease and mortality in general, evidence shows that it might not provide sufficient protection against virus transmission to prevent outbreaks of ND [37].

## Conclusion

We isolated and identified a viscerotropic velogenic strain of NDV that circulated among poultry farms in Diyala Province, Iraq. The techniques applied to identify the virus and its pathogenicity were straightforward, and RRT-PCR was the most sensitive. Vaccination programs using commercial virus vaccines did not confer a protective immune response against a virulent NDV isolate, indicating that vaccines should be prepared from circulating viruses using standard methods.

## Authors’ Contributions

AKA: Designed the study, editing of the manuscript and analysis. KSA: Editing of the manuscript and analysis. Both authors read and approved the final manuscript.
